# Preservation of Bilateral Corticospinal Projections from Injured Hemisphere After Perinatal Stroke

**DOI:** 10.3390/brainsci15010082

**Published:** 2025-01-17

**Authors:** Cameron P. Casey, Ellen N. Sutter, Alina Grimaldo, Kellie M. Collins, Jose Guerrero-Gonzalez, Ryan M. McAdams, Douglas C. Dean, Bernadette T. Gillick

**Affiliations:** 1 Waisman Center, University of Wisconsin-Madison, Madison, WI 53706, USAkellie.collins@wisc.edu (K.M.C.); jguerrerogon@wisc.edu (J.G.-G.); bgillick@wisc.edu (B.T.G.); 2 Department of Family Medicine and Community Health, University of Minnesota, Minneapolis, MN 55455, USA; 3 Department of Pediatrics, School of Medicine and Public Health, University of Wisconsin-Madison, Madison, WI 53706, USA; 4 Department of Medical Physics, School of Medicine and Public Health, University of Wisconsin-Madison, Madison, WI 53706, USA

**Keywords:** perinatal stroke, TMS, case report, motor development, corticospinal tract

## Abstract

Background: Perinatal brain injury is a leading cause of developmental disabilities, including cerebral palsy. However, further work is needed to understand early brain development in the presence of brain injury. In this case report, we examine the longitudinal neuromotor development of a term infant following a significant loss of right-hemispheric brain tissue due to a unilateral ischemic stroke. Our analysis focuses on the integrity and development of the corticospinal tract (CST) from the lesioned hemisphere. This case provides a unique opportunity to evaluate CST development after loss of the majority of the motor cortex. Methods: Evaluations were conducted when the infant was 4 (Visit-1), 18 (Visit 2), and 25 (Visit 3) months old. Assessments included magnetic resonance imaging (MRI) to characterize the lesion and quantify CST structural integrity, single-pulse transcranial magnetic stimulation (spTMS) to evaluate CST functional circuitry, and neuromotor assessments. Results: At Visit 1, bilateral CSTs were identified through diffusion-weighted MRI (dMRI) despite an estimated loss of 92.7% (7.3% retained) of age-typical motor cortex from the right hemisphere. Both hemispheres exhibited bilateral motor-evoked potential in response to stimulation with spTMS, which remained when reassessed at Visits 2 and 3. Longitudinal MRI showed distinct developmental trajectories of CST integrity in each hemisphere, with the lesioned hemisphere exhibiting initial increases in integrity between Visits 1 and 2 followed by a decrease in integrity between Visits 2 and 3. The non-lesioned hemisphere showed increased integrity from Visit 1 to Visit 2, which remained stable at Visit 3. Motor assessments at all visits indicated a high risk of cerebral palsy. Conclusions: This report highlights the utility of MRI and spTMS in studying neuromotor development. The findings reveal preserved functional bilateral CST circuitry despite majority loss of the right-hemispheric motor cortex as well as distinct developmental trajectories in CST integrity between hemispheres. These results underscore the potential for neural plasticity after perinatal brain injury. Clinical Trials Registration: NCT05013736.

## 1. Introduction

Perinatal brain injury encompasses a spectrum of neurological injuries occurring at or around the time of birth, leading to significant morbidity and long-term neurodevelopmental challenges [1]. These injuries are a leading cause of cerebral palsy (CP), and often result in motor deficits, cognitive impairments, and other neurological sequelae that persist into later life stages [2,3]. The first two years of life are critical for developing brain connectivity and motor function in infants, particularly those affected by perinatal brain injury [4]. This early period of rapid brain development provides a unique opportunity to study the mechanisms underlying motor function and the impact of early brain injuries on critical neural pathways. The corticospinal tract (CST), a major white matter pathway, is essential for voluntary motor control and the performance of skilled movements [5]. Damage to the CST during this period may disrupt the development of this circuitry, leading to motor deficits [6].

At birth, in typically developing infants, the CST projects bilaterally from each hemisphere. Ipsilateral projections are pruned over time, resulting in a predominance of contralateral fibers [6]. During the early years of life, myelination and neuronal pruning refine these motor pathways, establishing the foundation for neural communication that supports movement and coordination [7].

Early brain injuries, such as strokes or brain bleeds, may alter typical CST development, leading to retained ipsilateral projections from the less affected hemisphere [6,8]. The timing, location, and extent of injury have been identified as key factors in determining atypical neuromotor organization [9,10]. Early findings suggest that reduced contralateral control of the paretic limb correlates with poorer motor outcomes [11].

This case study presents a 2-year-old child with functionally intact bilateral CST projections, from both hemispheres, despite cortical involvement from a perinatal stroke. Structural and functional evaluations of the CSTs were performed using diffusion-weighted MRI (dMRI) and single-pulse transcranial magnetic stimulation (spTMS), respectively, providing insights into the neurologic underpinnings of retained ipsilateral circuitry and its functional implications. By presenting this example case, we aim to highlight how severe motor cortex damage may influence CST structure and function, offering a unique perspective on neuroplasticity during a critical developmental window. A deeper understanding of retained ipsilateral circuitry and neurodevelopmental mechanisms may help refine therapeutic approaches for early brain injury.

## 2. Methods

The child was enrolled in a longitudinal observational study of infants with perinatal brain injury (NCT05013736) and was evaluated according to our published protocol [4]. The assessments included MRI, spTMS, the General Movements Assessment (GMA) [12], and the Hammersmith Infant Neurological Examination (HINE) [13]. Detailed descriptions of the assessments are included below, while additional context regarding the overall study design can be found in the cited protocol [4].

Assessments were carried out at three key developmental stages: 4 months (Visit 1), 18 months (Visit 2), and 25 months (Visit 3) post-birth. According to our protocol, an additional visit around 12 months of age was planned; however, the participant was unable to attend due to an unrelated infection. The GMA, which assesses spontaneous movement patterns, was recorded at 16 weeks, 6 days post-term by the child’s parent following provided guidelines and later scored by two certified raters via recorded video. The GMA was scored as absent, sporadic, or normal fidgety movements and further analyzed for differences in the presence of fidgety movements between sides of the body. The HINE, which assesses infant muscle tone, cranial nerves, reflexes, and posture, was administered at all visits by a licensed physical therapist. HINE global scores and asymmetries were considered for analysis. At Visits 2 and 3, the participant’s mother completed a parent-report therapy survey designed to capture detailed information about the child’s therapeutic interventions. This survey collected data on the type of therapy received (physical therapy, occupational therapy, speech/language therapy, other, none), the frequency of therapy (<1 time per month, 1–2 times per month, 1 time per week, 2 times per week, >2 times per week), and the setting of therapy (in-home, outpatient clinic, daycare/school). Notably, this survey was not part of the study protocol at the time of Visit 1 and, therefore, data from that visit are unavailable.

MRI data were acquired at the University of Wisconsin-Madison on a 3-Tesla Discovery MR750 MRI scanner (GE Healthcare, Waukesha, Wisconsin). The imaging protocol included structural T1-weighted (T1w) and multi-b-value dMRI. The lesioned area was manually segmented on the T1w images using ITK-SNAP [14]. The UNC 1-year AAL parcellation atlas [15] was registered to the Visit-1 T1w scan using FLIRT [16] for affine registration, followed by ANTs diffeomorphic registration [17], providing an anatomically matched motor cortex (M1) mask. This mask was used to estimate M1 tissue loss [15] in the lesioned hemisphere M1, which was calculated as Volume (M1 mask—lesion mask)/Volume (M1 mask).

The dMRI data were preprocessed per our published protocol [4]. Diffusion tensor imaging, constrained spherical deconvolution (CSD), and neurite orientation dispersion and density imaging [18,19] models were fit to the preprocessed dMRI, using the Dipy (Version-1.9.0) [20], MRtrix3 (Version-3.0.4) [21], and Dmipy (Version-1.0.5) [22] software packages, respectively. TractSeg was used to segment white matter bundles and generate probabilistic tractograms for bilateral CSTs [23]. The mean of the non-diffusion-weighted images was affinely aligned to the MNI space using ANTs [17]. The resulting transform was applied to the CSD-based fiber orientation distribution (FOD) maps, which were included as part of the TractSeg workflow. Additionally, a more permissive threshold of 0.05 was allowed for initializing the region inside which probabilistic tracking was performed to deliver the final tract delineations. Delineated tractograms were mapped back into the subject space using the inverse affine transformation. Tract density images were computed and thresholded to remove voxels with a high cerebrospinal fluid (CSF) content (Isotropic Volume Fraction > 0.5) or voxels with a value of <5% of the robust maximum (98th percentile) of the streamline distribution. CST fractional anisotropy (FA) was used as an index of white matter integrity [24] with values calculated as weighted averages across the tract. Bootstrapped 95% confidence intervals (CIs) were computed with 10,000 iterations, and significant differences between tracts were determined based on non-overlapping CIs. FA values are reported as Mean [lower-CI, upper-CI].

A Magstim 200^2^ stimulator (Magstim, Whitland, UK) with a D70^2^ figure-of-eight coil was used for spTMS with simultaneous electromyography (EMG) recording from bilateral wrist flexor muscles. T1w images were used for neuronavigation in Brainsight software (Version-2.4.11, RRID:SCR_009539, Rogue Research, Montreal, QC, Canada) to stimulate the putative hand knob region of M1. For the lesioned hemisphere, where no identifiable hand knob was present, the stimulation target was placed proximal to the remaining cortical tissue across the longitudinal fissure from the contralateral M1. Stimulation intensity began at 50% maximum stimulator output (MSO) and increased by increments of 5% until motor-evoked potentials (MEPs) could be consistently evoked or until 80% MSO was reached. A minimum of 10 s was allowed between pulses. MEPs were determined based on visual examination of EMG recordings, with consensus from three experienced TMS researchers (CPC, ENS, BTG).

### Standard Protocol Approvals, Registrations, and Patient Consents

The data were collected under an IRB-approved protocol (2021-0412) at the University of Wisconsin-Madison. Informed written consent for data collection and dissemination was obtained from the child’s parents as part of the study enrollment.

## 3. Results

This female term infant was enrolled in a longitudinal observational study [4] following a right-hemispheric ischemic stroke that occurred4 weeks after birth (gestational age: 38 weeks 3 days). Clinical MRI at the time of the injury demonstrated restricted diffusion and T2 hyperintensity throughout the right cerebral hemisphere and right lateral ventricular dilation.

### 3.1. Visit 1 (4 Months)

**Figure 1:**
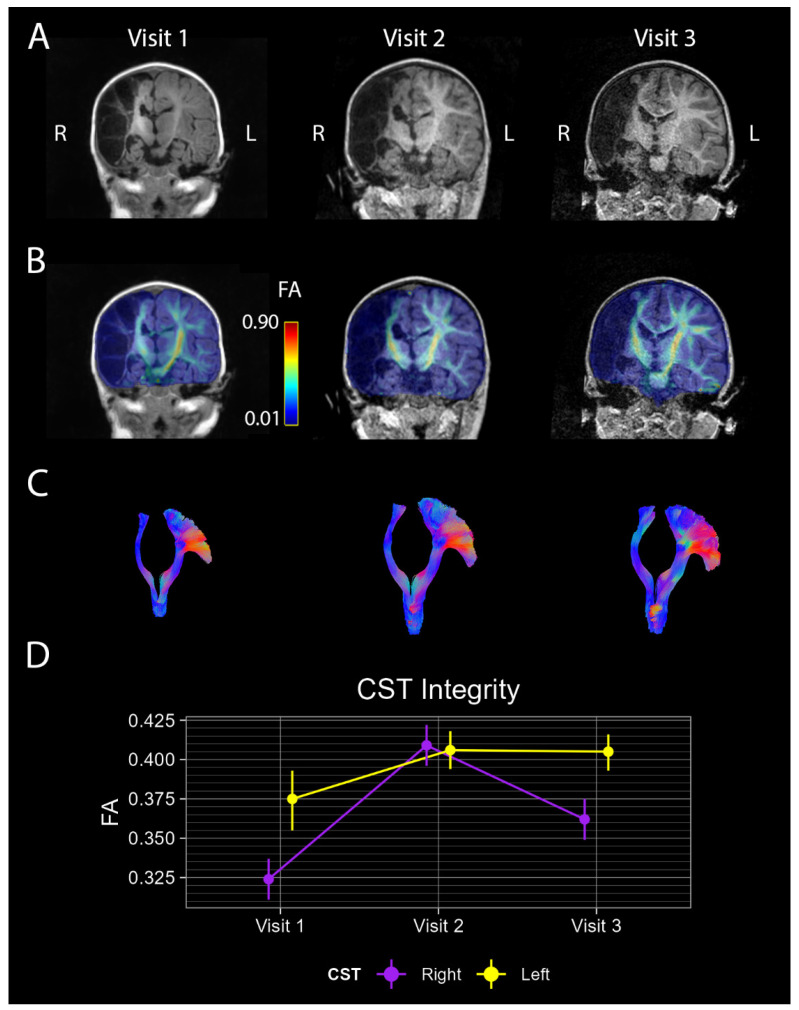
MRI characterization of brain injury and CST integrity across visits. (**A**). Coronal views of T1w images from Visit 1 (**left**)**,** Visit 2 (**middle**), and Visit 3 (**right**). The majority of the right hemisphere has been replaced by cerebrospinal fluid following perinatal ischemic stroke. (**B**). FA maps derived from processed dMRI scans overlaid over T1w images from (**A**). CSTs are evidenced in left and right hemispheres as warmer colored regions between cortex and brainstem. (**C**). CST tract images generated by Tractseg. CSTs were identified in both hemispheres, matching with the higher FA regions shown in (**B**). Color mapping shows the orientation of the white matter bundles (red—left-right, green—anterior-posterior, blue—inferior-superior) (**D**). Quantification of FA values from right (purple) and left (yellow) CSTs at both study visits. Dots indicate the weighted-mean FA while lines indicate the bootstrapped 95% CI of the weighted mean.

At 4 months (Visit 1), research MRI scans showed extensive loss of right-hemispheric brain tissue and ventricular dilation (Figure 1A left), consistent with prior clinical scans. The right M1 was estimated to retain only 7.3% of its expected volume, based on the registered UNC 1-year-old AAL parcellation. Despite this tissue loss, diffusion-imaging revealed preserved bilateral CSTs (Figure 1B left), which were mapped via tractography (Figure 1C left). Weighted-mean FA values were 0.375 [0.355, 0.393] for the left CST and 0.324 [0.311, 0.337] for the right CST, indicating reduced integrity on the lesioned side (Figure 1D).

Motor assessments performed at Visit 1 showed atypical development. The GMA showed absent fidgety movements on the left side of the body with sporadic fidgety movements on the right, indicating a high risk of developing cerebral palsy (CP) [12]. The child achieved a HINE Global Score of 49, indicating high risk of unilateral CP given the age-appropriate cut-off score of 56 and the presence of >5 asymmetries [13,25,26].

**Figure 2:**
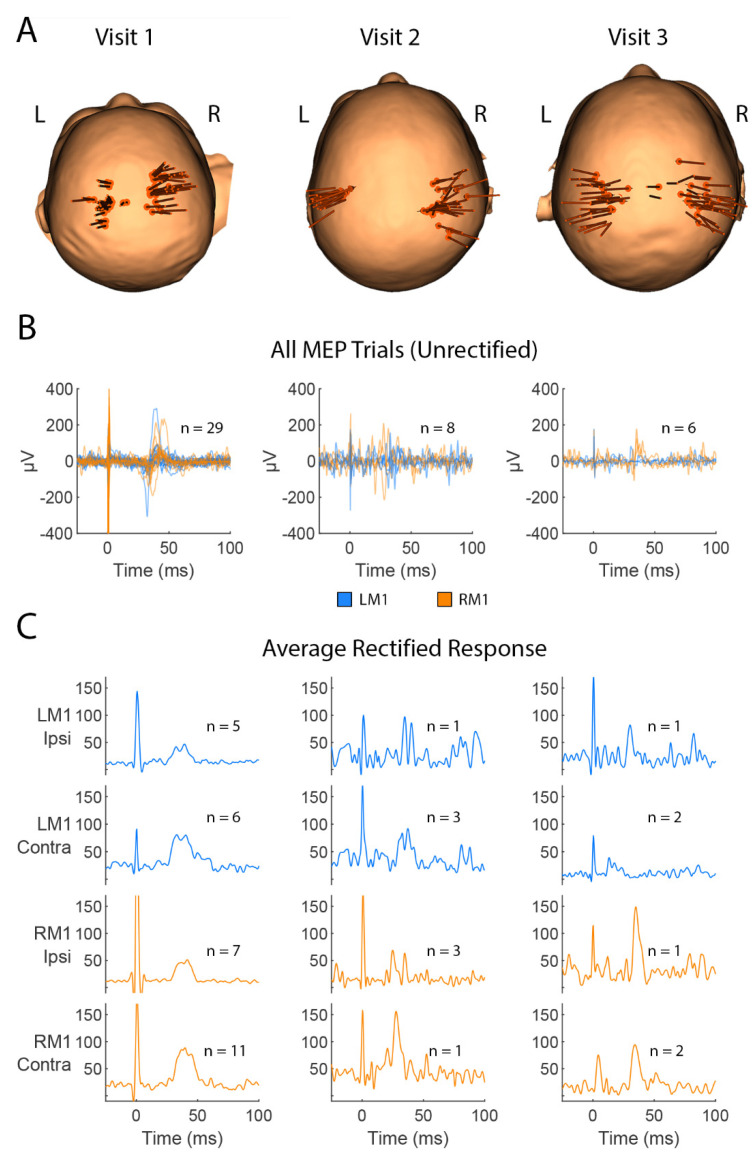
TMS assessment and MEP responses. (**A**). TMS stimulation trajectories (orange arrows) indicating the location and tilt angle of the coil for each trial at Visit 1 (**left**), Visit 2 (**middle**), and Visit 3 (**right**). Images depict a top-down view of the child’s scalp as if standing behind them. (**B**). EMG recordings from 25 ms before the TMS pulse to 100 ms after the pulse showing trials in which MEPs were identified during Visit 1 (**left**), Visit 2 (**middle**), and Visit 3 (**right**). Time 0 indicates when the TMS pulse was delivered. EMG was collected at 3000 Hz, high-pass filtered at 10 Hz, and baseline-centered prior to plotting. Trials in which stimulation was applied to the left hemisphere are shown in blue while right hemisphere trials are shown in orange. (**C**). Rectified EMG for trials in which MEPs were identified, averaged by target (left-M1; LM1 or right-M1; RM1) and the recorded hand (ipsilateral hand; Ipsi or contralateral hand; Contra) for Visit 1 (**left**) and Visit 2 (**right**). Each subplot is labeled with the number of averaged trials. Prior to averaging, EMG traces were rectified to make all values positive and then smoothed with a peak-to-peak envelope to attenuate noise from zero-crossings. This procedure aids in visual identification of consistent changes in EMG amplitude without assuming intertrial phase consistency. The presence of MEP signals can be seen as peaks emerging 25–40 ms after the TMS pulse. MEP presence indicates a functional connection between the stimulated brain region and recorded muscle. Y-axes are in units of μV.

spTMS was performed on both hemispheres using an intensity range of 50–75 MSO. A total of 31 pulses were applied to the left hemisphere and 39 to the right hemisphere (Figure 2A left). MEPs were observed in both wrist-flexors from stimulation of both hemispheres, indicating functionally intact bilateral CST circuitry from both hemispheres (Figure 2B,C left). At 6 months of age, the child was diagnosed clinically with left hemiparesis and gross motor delay.

### 3.2. Visit 2 (18 Months)

At the 18-month visit (Visit 2), T1w MRI showed expected developmental differentiation between gray and white matter without notable changes in brain injury characteristics (Figure 1A middle). The FA map and tractography confirmed the persistence of bilateral CSTs, with weighted-mean FA values of 0.406 [0.394, 0.418] and 0.409 [0.396, 0.422] for the left and right CST, respectively (Figure 1B–D middle). The child’s HINE Global Score increased to 56, with 18 asymmetries identified, indicating a continued high risk of unilateral CP given the age-appropriate cut-off score of 65 and the presence of >5 asymmetries [13,25,26]. The parent-report therapies survey indicated that the participant was receiving physical, occupational, and speech/language therapy, each with a frequency of >2 times per week. Physical and occupational therapies were conducted both at in-home and at outpatient clinical settings, while speech/language therapy was provided exclusively at home.

spTMS was conducted using intensities ranging from 50 to 80% MSO. A total of 17 pulses were delivered to the left hemisphere and 25 pulses to the right (Figure 2A middle). MEPs were again observed in both arms in response to stimulation of both hemispheres (Figure 2B,C middle), confirming continued bilateral functionality of the CSTs.

### 3.3. Visit 3 (25 Months)

At the 25-month visit (Visit 3), research MRI showed maintained bilateral CSTs, though FA values for the right CST declined slightly to 0.362 [0.349, 0.375], while the left CST remained stable at 0.405 [0.393, 0.416] (Figure 1B–D right). The child’s HINE Global Score was 58, with 14 asymmetries identified, continuing to indicate a high risk of unilateral CP based on the age-appropriate cut-off score of 65 and the presence of >5 asymmetries [13,25,26]. According to the parent-report therapies survey, the participant continued to receive physical, occupational, and speech/language therapy, each with a frequency of >2 times per week. All therapies were conducted in both home and outpatient clinical settings.

spTMS was completed with 31 pulses delivered to the left hemisphere and 26 pulses delivered to the right (Figure 2A right), at intensities of 50–75% MSO. MEPs were detected in both wrist-flexors following stimulation of both hemispheres, further supporting intact bilateral CST functionality (Figure 2B,C right).

No adverse events occurred across all visits.

## 4. Discussion

This report identified preserved bilateral CST projections in a child who experienced extensive right-hemispheric motor cortex loss from a perinatal stroke, eliminating an estimated 92.7% of the motor cortex in the lesioned hemisphere. Four months after birth, the FA of the right CST was significantly lower than that of the left, but by 18 months, FA values for both tracts increased and became comparable. By 25 months, the FA of the right CST declined, suggesting distinct developmental trajectories influenced by the injury.

While structural CST changes were evident through dMRI, we observed the same pattern of functional circuity at all visits using spTMS, with both hemispheres bilaterally projecting to both wrist-flexors. This circuitry pattern is typical at birth. However, ipsilateral spTMS responses in distal arm muscles after 18 months are rarer [27]. Ipsilateral CST pruning is thought to be facilitated by competitive activation [27], thus the retention of bilateral projections from the injured hemisphere may indicate a degree of continued motor control from this hemisphere. Loss of CST projections from the injured hemisphere may occur after perinatal brain injury [8]. The persistent bilateral responses from the lesioned hemisphere, observed via spTMS, are unexpected and suggest sustained motor control capabilities from the injured hemisphere. However, the clinical significance and persistence of these bilateral projections remain unclear. The observed decline in FA of the right CST at 25 months may indicate withdrawal of contralateral projections from the lesioned hemisphere (i.e., ipsilesional withdrawal), a process proposed as a mechanism for the development of ipsilateral CST circuitry [27]. The decrease in FA on the lesioned side may therefore represent this withdrawal process. However, since MEPs were still elicited from the lesioned hemisphere, this putative withdrawal process cannot be considered complete. Further withdrawal may occur, and MEPs from the injured hemisphere could potentially diminish over time. A follow-up study would be necessary to fully characterize the final structure and functional organization of the CST in this child.

It is worth noting that while MEPs were observed in both wrist-flexors in response to stimulation of the left and right hemisphere at all visits, the number of MEPs observed was reduced at Visits 2 and 3 relative to Visit 1 (Figure 2B,C). This may be partially due to a decrease in the number of TMS pulses applied during the later sessions; 70 pulses were delivered at Visit 1, while 42 were delivered at Visit 2 and 57 at Visit 3. However, if this were the only explanation, we would still expect the proportion of pulses that resulted in an MEP to remain roughly the same across visits. The percentage of pulses that resulted in an MEP was 33%, 17%, and 11% at Visit- 1, 2, and 3, respectively. The decrease in the proportion of pulses that resulted in an MEP, relative to the total pulses delivered, at Visits 2 and 3 suggests that the total number of pulses does not fully explain the lower number of MEPs at the later visits. The number of observed MEPs may also be related to changes in child behavior throughout development. As children grow and develop volitional motor skills, they also may become more mobile and eager to exert their independence [28]. This mobility can pose challenges for spTMS assessment, which depends upon accurate coil positioning over the motor cortex, a task that becomes much more difficult when participants are moving. To address these challenges, we make use of age-appropriate toys, books, and videos to hold participant attention and reduce movement during spTMS sessions; however, these strategies do not prevent motion entirely. It is also possible that the decrease in observed MEPs is related to changes in CST excitability, yet due to the competing explanations already stated, we refrain from making this inference.

An important limitation of this study is the scarcity of normative data that can be used for comparison. While there have been a few studies examining CST circuitry and development in infants [27,29,30,31], small sample sizes limit the generalizability of these findings as a norm reference (see [32] for a review of infant TMS studies). There are also no published data evaluating the development of CST circuitry to wrist flexor muscles in typically developing infants. Because of this, we are limited in the conclusions we can draw regarding what is expected for a child at each time point in this study, particularly in the presence of a brain injury. However, we posit that the observed retention of bilateral circuitry from the lesioned hemisphere is atypical with regards to what little has been published from this age range [27,29]. This limitation also underscores the importance of further longitudinal studies to better characterize the variability of CST development in infants with perinatal brain injuries.

The potential influence of therapeutic interventions outside the scope of this study on the participant’s development should also be considered. The parent-report therapies survey indicated that the participant received frequent (>2 times per week) physical, occupational, and speech/language therapy services during Visits 2 and 3 (data not available for Visit 1). Early intervention, including physical therapy, has proven efficacious in improving motor outcomes for infants with perinatal brain injury [33]. Some research indicates that these motor improvements come with neuroanatomical changes measurable by MRI [34]. It is plausible that the data presented here were influenced by the therapies the participant engaged in as part of routine clinical care. For example, the HINE scores may have been higher than they would have been in the absence of intervention. Similarly, the retention of functional CST projections from the lesioned hemisphere at 2 years of age might have been facilitated through therapy-related engagement of these upper motor neurons. However, the degree to which the results were influenced by therapeutic interventions cannot be parsed from this single case. Standard care therapies are particularly challenging to control experimentally considering the ethical issues of withholding a treatment of known benefit. Future research should focus on developing methods to statistically disentangle the interactions between therapeutic interventions and neurodevelopmental outcomes following perinatal brain injury. 

Movement assessments at all visits indicated a high risk of CP. Although this child was diagnosed with left hemiplegia and gross motor delay, no formal CP diagnosis was made. The clinical reasoning for the specific diagnoses that were or were not provided is not known. However, recent work has shown that CP diagnosis strategies between physicians are variable [35] and some providers are hesitant to provide a CP diagnosis before the age of 2 years [36].

## 5. Conclusions

This case highlights the remarkable neuroplasticity of the infant brain, demonstrating functional connectivity retention despite extensive neuronal damage. The use of dMRI and spTMS was instrumental in delineating these adaptive processes, highlighting their potential utility in tracking neurodevelopmental outcomes after perinatal brain injury.

## Data Availability

The data for this case report are not publicly available beyond what is published in the main text. The data may be made available upon reasonable request with data sharing approval from the IRB. The data are not publicly available due to protected health information and participation in an ongoing study.
